# Long-Term Complications of Retained Gallstones After Minimally Invasive Cholecystectomy: Hidden Perils

**DOI:** 10.7759/cureus.79645

**Published:** 2025-02-25

**Authors:** Syona Tuladhar, Bernard Zaragoza

**Affiliations:** 1 Department of Surgery, Nova Southeastern University Dr. Kiran C. Patel College of Osteopathic Medicine, Fort Lauderdale, USA; 2 Department of Surgery, Broward Health Coral Springs Medical Center, Coral Springs, USA

**Keywords:** abscess, dropped gallstones, lumbar hernia, minimally invasive cholecystectomy, retained gallstones

## Abstract

Minimally invasive cholecystectomy (MIC) is the preferred procedure for the treatment of acute cholecystitis. A common intraoperative complication includes gallbladder perforation with gallstone spillage into the peritoneal cavity. Though retained patients with gallstones are generally asymptomatic, they can, on rare occasions, present with complications months to years later. A wide range of complications, ranging from abscess formation to more unusual sequelae such as lumbar hernias, is possible. We present two atypical cases of retained gallstones following MIC to highlight the need for recognition of this complication. The first case is of a 43-year-old male who presented with persistent right upper quadrant pain six months post-laparoscopic cholecystectomy. A computed tomography (CT) scan revealed three gallstones as the nidus of infection. The second case is of a 78-year-old female who presented with recurrent intra-abdominal abscesses, eventually requiring a right-sided lumbar hernia repair, resulting from her retained gallstones.

## Introduction

Cholecystectomy is one of the most frequently performed surgeries in America, with approximately 1.2 million surgeries performed annually [1]. Since the introduction of laparoscopic surgery in the late 1980s, the gold standard of cholecystectomies has shifted from an open technique to a minimally invasive approach, with 92% of all cholecystectomies being performed laparoscopically [1]. As with any surgical procedure, it is crucial to recognize both intraoperative and postoperative complications that may occur. Frequently reported complications include gallbladder perforation, occurring in 10-40% of cases, and stone spillage, occurring in 6-30% of cases [2].

Many patients remain asymptomatic, with only 0.08-0.3% experiencing complications because of the spilled stones [2-3]. Of those, the most frequently reported complication from the spilled stones is the risk of fistula and abscess formation at various sites, such as the abdominal wall, subhepatic, and retroperitoneum. In rare instances, further superior migration into the pleural cavity can cause thoracic complications, including pleural empyema and cholelithopthysis [4]. Antibiotics or percutaneous drainage may be used as a temporary treatment for abscesses secondary to dropped gallstones. Percutaneous drainage offers a minimally invasive way to drain small calculi and accessible intrabdominal abscesses [5]. However, unlike other abdominal abscesses, until the source of infection (i.e., the dropped stone) is surgically removed, recurrence of the abscess is highly likely [6-7]. We present two cases to highlight the importance of a timely diagnosis and surgical evacuation of stones. Delays in proper diagnosis can lead to further complications and prolonged recovery after minimally invasive cholecystectomy (MIC).

## Case presentation

Case 1

**Figure 1:**
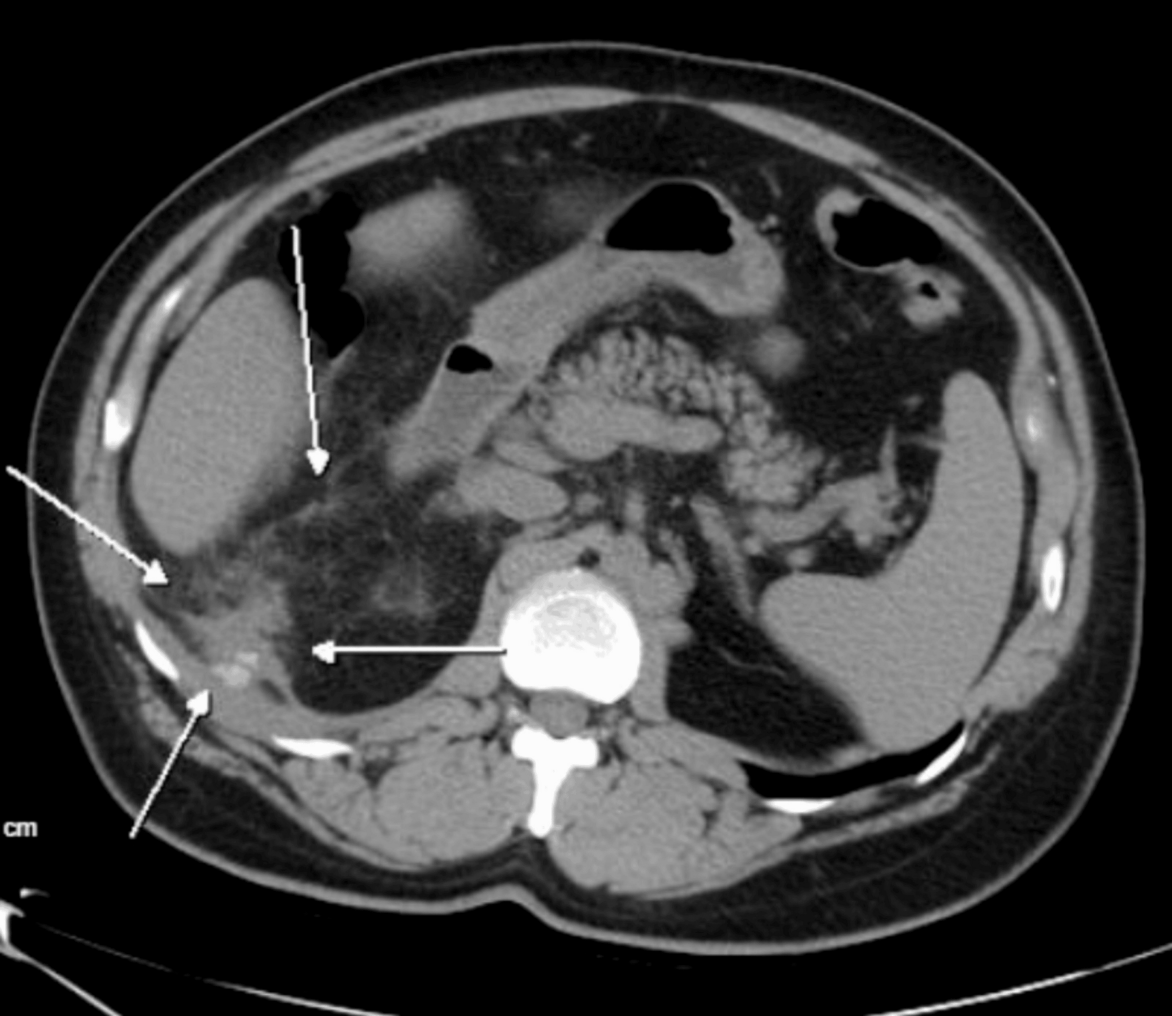
CT scan of the abdomen and pelvis demonstrating two hyperdense foci within the hyperdense area along the inferior margin of the right hepatic lobe. CT, computed tomography

A 43-year-old male presented to the clinic with night sweats and persistent right upper quadrant (RUQ) pain radiating to the ipsilateral flank six months following an elective laparoscopic cholecystectomy (LC). On physical examination, there was notable tenderness and erythema of the right flank. CT scan showed an ill-defined dense region posterior along the inferior margin of the right hepatic lobe, measuring 3.4 x 3cm with two hyperdense foci within the dense region measuring 9 mm and 6 mm, likely indicated as dropped gallstones (Figure 1). The patient completed multiple courses of antibiotics with temporary relief of symptoms.

**Figure 2:**
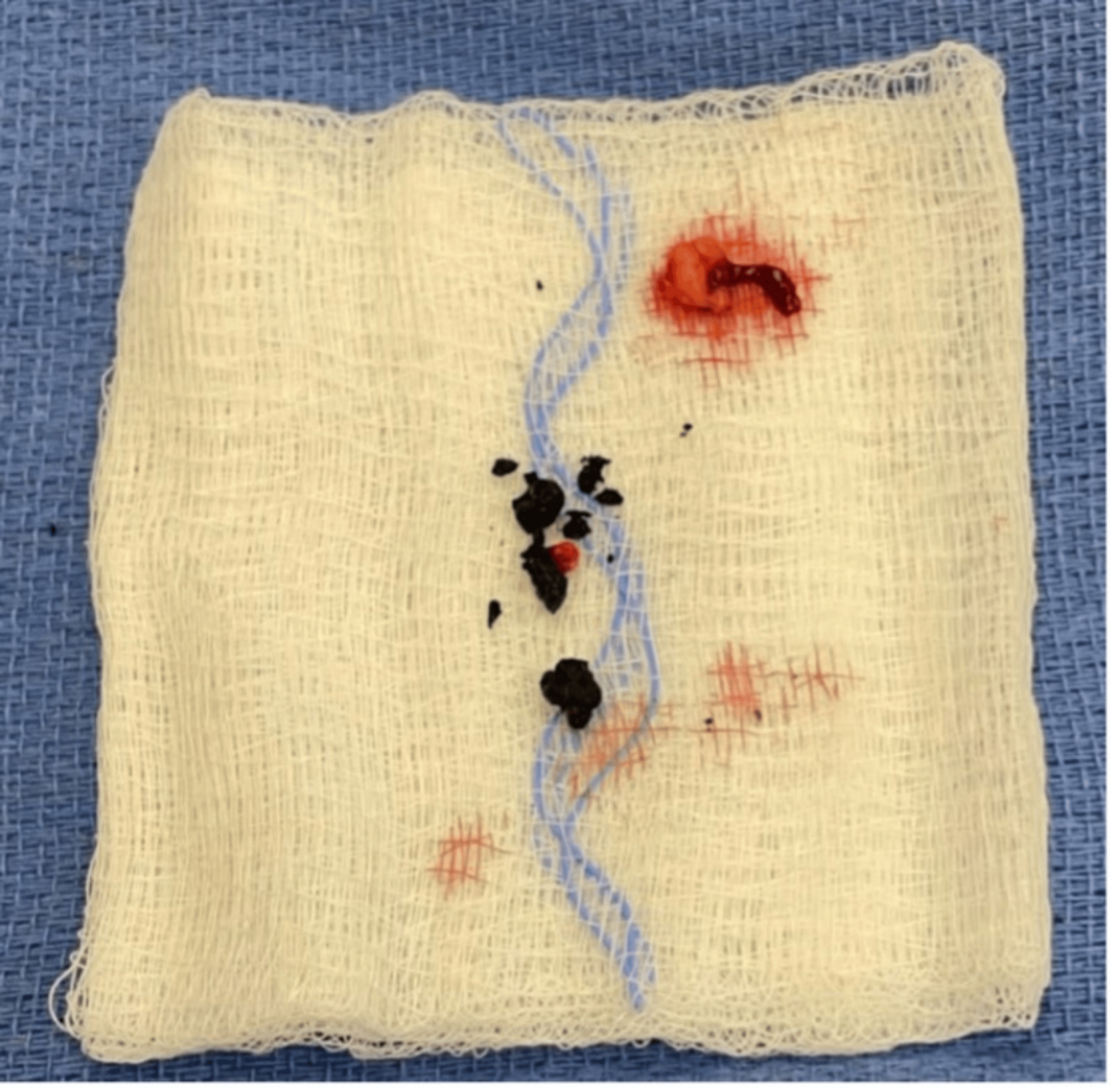
Retrieved fragments of the gallstones

The patient then underwent diagnostic laparoscopy with drainage of the abscess and evacuation of the retained gallstones approximately seven months after LC. Intraoperatively, the abscess cavity was localized and accessed just above the hepatic flexure and at the lower edge of the liver. The abscess appeared to be eroding into the abdominal wall. Additionally, evidence of adhesions related to the prior surgery was noted in the RUQ. A total of three gallstones were visualized and extracted. Once adequately cleaned, a 15F round BLAKE drain was placed in one of the 5-mm port sites. Pathology indicated multiple irregular jagged fragmented black crystalline choleliths measuring 1 x 1 x 1.07 cm in aggregate (Figure 2). Of note, the patient returned two months later to the clinic with persistent pain and cellulitis of the flank. CT scan revealed inflammation of the abdominal wall without evidence of stones or abscess formation. The patient was managed with oral antibiotics without further complications.

Case 2

**Figure 3:**
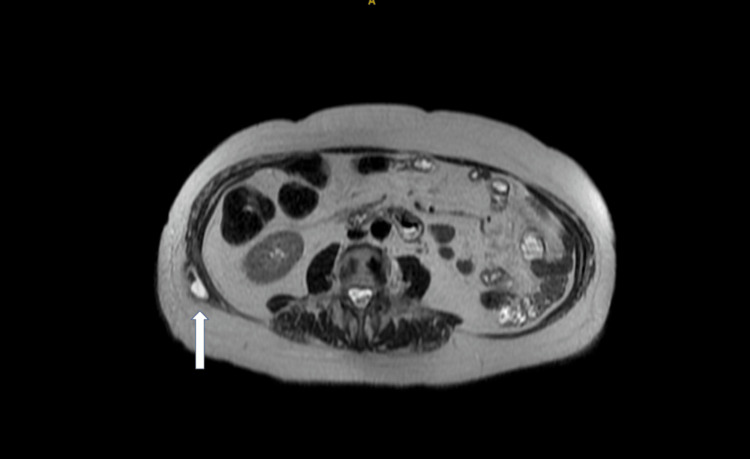
MRI showing 3 x 1 cm fluid collection in the right oblique muscle MRI, magnetic resonance imaging

A 78-year-old female initially presented in May 2019 with a history of cholecystitis with hepatic abscess, which was treated initially with a cholecystostomy tube. A subsequent cholecystectomy with cholangiogram was completed two months later. The patient presented to the emergency department two years later with complaints of right lower back pain, with imaging revealing the presence of repeat intra-abdominal abscesses. She denied fever, abdominal pain, or weight loss at that time. An ultrasound-guided interventional radiology (IR)-guided drainage was placed in the RUQ, but there was no significant drain output. The patient was asymptomatic at this time, but magnetic resonance imaging (MRI) was performed for further clarification, which revealed a 3 x 1 cm fluid collection in the right oblique muscle with adjacent fat induration (Figure 3). The patient underwent CT-guided drainage by IR in December 2022, and 7 mL of cloudy yellowish-red fluid from a right flank abdominal wall abscess was extracted; however, no drain was placed at that time, and cultures were negative. No intraoperative complications were noted. Unfortunately, the patient returned one month later in January 2023 with continued complaints of discomfort and swelling at the right flank but denied abdominal pain, fever, or nausea. An ultrasound-guided incision and drainage (I&D) of a right flank abscess was completed, during which a gallstone was identified and successfully removed. Despite tolerating the procedure well, the patient had continued swelling in the right flank and a notable lumbar bulge increasing in size with cough at the prior I&D site approximately four months later.

**Figure 4:**
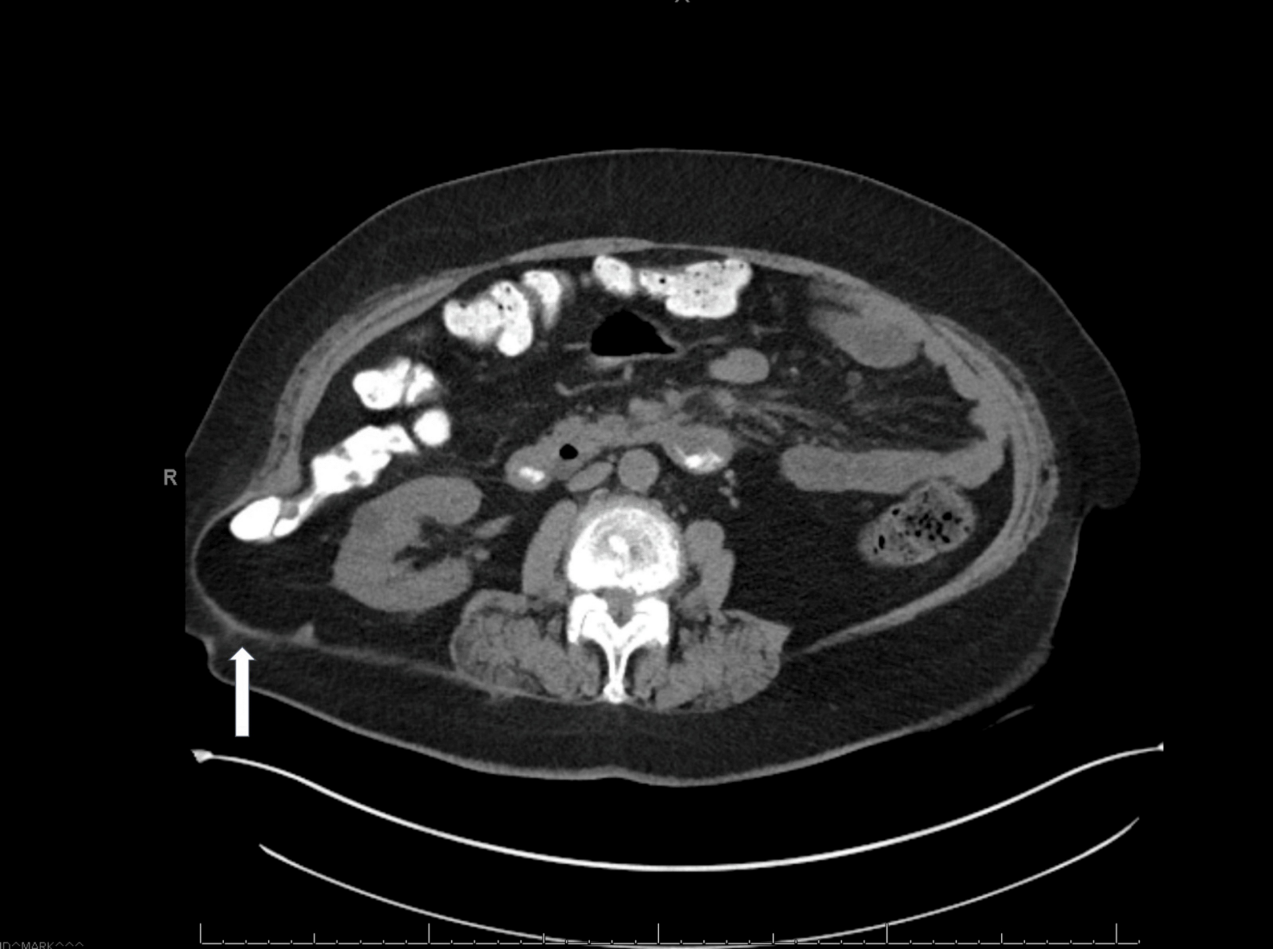
CT scan of the abdomen showing a hernia in the right posterior lumbar region CT, computed tomography

A CT scan confirmed the presence of a 10.9 x 6.5 cm right flank hernia containing fat, soft tissue, and a portion of the middle pole of the right kidney located at the previously queried abdominal wall abscess (Figure 4). There was no evidence of bowel obstruction. The patient underwent a robotic right lumbar incisional hernia repair with mesh in October 2023. The patient tolerated the procedure well and by nine months post-operatively showed improvement with no evidence of hernia recurrences or complications.

## Discussion

MIC is the gold standard for treating symptomatic cholelithiasis by offering less pain at incision sites, shorter hospital stays and recovery periods, lower morbidity and mortality, and enhanced quality of life compared to open surgery [8]. However, common intraoperative complications that may arise include gallbladder perforation, bile leakage, and bleeding, with the estimated incidence of gallbladder perforation varying from 10% to 40% [9-11]. Acute cholecystitis, obesity, diabetes, and male sex have been determined to be significant predictors of intraoperative complications [11].

In a review examining 18,280 LCs, the reported incidence of spilled stones was 7.3% and that of unretrieved peritoneal gallstones was estimated to be 2.4% [9]. There has been much discussion on strategies to reduce the risk of gallstone spillage, which include careful dissection of the gallbladder and the use of retrieval bags [12]. However, ultimately, every attempt by the surgeon should be made to retrieve the spilled stones via suctioning, forceps, and irrigation [13]. If such attempts are unsuccessful, it is imperative that surgeons thoroughly document and clearly communicate with patients regarding potential complications. An incomplete surgical note can lower the index of suspicion, hindering the timely diagnosis of dropped gallstones. Therefore, reporting these dropped gallstone events is crucial so that a high index of suspicion can be maintained and can positively impact treatment outcomes [14].

Complications from retained gallstones are rare and therefore have minimally been documented in the literature. Intra-abdominal abscesses account for 60% of these complications and commonly manifest within the peritoneal cavity, at port sites, or in the thorax [10]. Previous reports have shown that stones may travel to unusual locations in the body causing widespread complications such as pleural effusion, broncholithiasis, splenic abscess, infertility, chronic pelvic pain, intraluminal bowel obstruction, and incarcerated inguinal hernia [4,15-19]. The unusual location of the retained stone forming distant abscesses, such as in the right flank as in our case two can delay identification of retained gallstone as the etiology.

Diagnosis of retained gallstones is further challenged due to the vague presentation of symptoms along with a low index of suspicion by physicians [5]. Patients typically present with abdominal pain, fever, weight loss, and nausea or vomiting, as was observed in case 1. The onset of symptoms can also vary with the average time of presentation being four to five months after MIC, although symptoms can also appear as early as a few weeks post-operatively or even years after [9,20]. In our case 2, the patient presented with symptoms within three months of LC, while case 2 presented with more unusual symptoms (i.e., lower back pain) two years later.

The definite treatment of abscesses caused by retained gallstones involves pus drainage and complete removal of stones. Percutaneous ultrasound-guided drainage with saline irrigation can be an effective method to treat superficial abscesses containing small calculi [5]. For deep abscesses involving stones larger than 1 cm, percutaneous procedures can be used but success in those cases is more variable. These stones most require fragmentation via ultrasonic lithotripsy or rigid endoscopy [20]. However, without actual stone removal, antibiotic treatment and drainage will not be enough to prevent recurrent abscesses [5]. In both cases, our patients underwent antibiotic treatment for their abscess with no relief. However, in case 2, ultrasound-guided percutaneous methods were also used to drain the right flank abscess. While gallstone evacuation was successful, unforeseen complications, such as a right-sided lumbar hernia, emerged four years later thus further delaying recovery.

## Conclusions

Retained gallstones following LC, though uncommon, can present as a significant clinical challenge. We highlight the importance of comprehensive documentation and informing patients when gallstones are retained regardless of how small the risk of potential complications is. Clinicians should remain vigilant and maintain a high index of suspicion in patients with a past surgical history of cholecystectomy who present with ongoing non-specific symptoms. The variability in presentation without definite cause highlights the need for individualized care to optimize recovery outcomes. Ultimately, in both cases, complete evacuation of stones successfully resolved the patient’s symptoms. While percutaneous drainage and antibiotics serve as useful first steps in management, they should be regarded as temporary measures rather than definitive treatment. Removal of all retained gallstones is essential to ensure complete resolution of symptoms and prevent recurrence.
